# Synthesis of Silver Phosphate (Ag_3_PO_4_) and Its Supplementation to Enhance Phytochemical Accumulation and Antioxidant Activity of *Cryptocoryne albida* Under Hydroponic Cultivation

**DOI:** 10.3390/ijms27146235

**Published:** 2026-07-13

**Authors:** Somkiat Seesanong, Banjong Boonchom, Chaowared Seangarun, Nongnuch Laohavisuti, Benyakorn Labantao, Pesak Rungrojchaipon, Sirichet Punthipayanon, Wimonmat Boonmee

**Affiliations:** 1 Office of Administrative Interdisciplinary Program on Agricultural Technology, School of Agricultural Technology, King Mongkut’s Institute of Technology Ladkrabang, Bangkok 10520, Thailand; somkiat.se@kmitl.ac.th (S.S.); 65046021@kmitl.ac.th (B.L.); 2 Material Science for Environmental Sustainability Research Unit, School of Science, King Mongkut’s Institute of Technology Ladkrabang, Bangkok 10520, Thailand; chaowared@gmail.com (C.S.); pesak.ru@kmitl.ac.th (P.R.); sirichet@g.swu.ac.th (S.P.); 3 Municipal Waste and Wastewater Management Learning Center, School of Science, King Mongkut’s Institute of Technology Ladkrabang, Bangkok 10520, Thailand; 4 Department of Chemistry, School of Science, King Mongkut’s Institute of Technology Ladkrabang, Bangkok 10520, Thailand; 5 Department of Sports Science, Faculty of Physical Education, Srinakharinwirot University, Bangkok 10110, Thailand; 6 Department of Biology, School of Science, King Mongkut’s Institute of Technology Ladkrabang, Bangkok 10520, Thailand; wimonmat.bo@kmitl.ac.th

**Keywords:** silver phosphate, hydroponics, aquarium plant, *Cryptocoryne albida*

## Abstract

This study investigated the supplementation of silver phosphate (Ag_3_PO_4_) to enhance the growth and antioxidant activity of *Cryptocoryne albida* in a hydroponic system. Ag_3_PO_4_ was synthesized via a simple precipitation method using AgNO_3_ and (NH_4_)_2_HPO_4_ as precursors, with alcohol employed as the reaction medium to control particle growth and agglomeration. FTIR analysis confirmed the presence of characteristic phosphate (PO_4_^3−^) functional groups, while XRD revealed diffraction peaks consistent with Ag_3_PO_4_ as the dominant crystalline phase. The SEM image exhibits relatively uniform spherical particles of 300–500 nm. Moreover, the XRF results for the synthesized Ag_3_PO_4_ indicated 86.80% of Ag_2_O and 12.60% of P_2_O_5_ as the major components. The application experiment was conducted in a DFT hydroponic system containing different concentrations of Ag_3_PO_4_-derived supplementation (0.00, 0.01, 0.10, and 1.00 ppm). The highest concentration (1.00 ppm) of Ag_3_PO_4_ in the KMITL2 nutrient solution significantly reduced plant growth (*p* < 0.05). In contrast, Ag_3_PO_4_-derived supplementation with 0.01 ppm resulted in significantly higher phytochemical accumulation, including total phenolic content (TPC), total flavonoid content (TFC), and antioxidant activities evaluated by DPPH and ABTS assays (*p* < 0.05). These results suggest that low-concentration Ag_3_PO_4_-derived supplementation may promote phytochemical accumulation and antioxidant activity in *C. albida*. Overall, this study highlights the potential of Ag_3_PO_4_-derived supplementation for modulating phytochemical accumulation and antioxidant activity in hydroponically cultivated *C. albida*.

## 1. Introduction

Currently, aquatic plants are important economic crops with strong international demand. Thailand exports aquatic plants to more than 70 countries worldwide, and over 250 species are commonly used in aquaria due to their ornamental value and adaptability [1]. Among them, *Cryptocoryne albida* Parker is a promising species for commercial production and export [2]. Native to southern Thailand, this species adapts well to aquatic and hydroponic environments, making it suitable for controlled cultivation systems [3,4]. Several *Cryptocoryne* species have been successfully cultivated hydroponically for commercial purposes [2]. In addition to growth performance, the commercial value of aquatic plants is influenced by their physiological and biochemical characteristics. Plant physiological and biochemical responses can be influenced by supplemental materials added to hydroponic systems. However, their effects are often concentration-dependent, and excessive exposure may result in phytotoxicity [5,6,7]. Silver phosphate (Ag_3_PO_4_) is a silver-containing phosphate material with unique physicochemical properties, but information regarding its effects on aquatic plants remains limited. Therefore, the effects of Ag_3_PO_4_ on plant physiological and biochemical responses remain of considerable interest. In particular, its influence on growth, chlorophyll content, phytochemical accumulation, and antioxidant activity in *C. albida* under hydroponic cultivation conditions warrants further investigation.

Previous studies have shown that low concentrations of silver-based materials, particularly silver nanoparticles (AgNPs), can influence plant growth, phytochemical accumulation, and antioxidant responses in a concentration-dependent manner. Such effects have been associated with hormetic responses, in which low levels of stress stimulate beneficial physiological and biochemical processes, whereas excessive exposure may induce phytotoxicity. Experimental studies have reported enhanced plant growth, antioxidant activity, and secondary metabolite accumulation following treatment with low concentrations of AgNPs, although the magnitude and direction of the responses vary among plant species and cultivation conditions [8,9,10]. These findings suggest that silver-containing materials may serve as potential elicitors for modifying plant physiological and biochemical characteristics when applied at appropriate concentrations. Our previous study demonstrated the successful synthesis of spherical Ag_3_PO_4_ particles through a simple and rapid precipitation method and evaluated their application in a plant tissue culture system [11]. The results showed that Ag_3_PO_4_-derived preparation could be applied in plant culture systems without obvious adverse effects under the tested conditions, highlighting its potential for further investigation in plant cultivation applications [11]. In addition, phosphate ions may provide supplementary nutrients for plant growth when applied at appropriate concentrations, which is particularly beneficial for hydroponic and aquatic plant cultivation systems [12,13]. However, excessive exposure to silver-containing materials may result in phytotoxic effects, including growth inhibition, chlorophyll reduction, and oxidative stress [14]. Therefore, optimization of Ag_3_PO_4_ concentration is essential to maximize beneficial plant responses while minimizing adverse effects. Despite these observations, information regarding the effects of Ag_3_PO_4_ on aquatic plants cultivated under hydroponic conditions remains limited.

Silver phosphate is commonly synthesized via a simple aqueous precipitation method, in which a soluble silver salt, such as silver nitrate (AgNO_3_), is reacted with a phosphate source, including sodium phosphate, potassium phosphate, or ammonium phosphate [11,15]. In our previous study, a simple and rapid synthesis of spherical Ag_3_PO_4_ with pronounced antibacterial activity was successfully demonstrated in plant tissue culture systems, which revealed that the choice of phosphate precursor plays a critical role in determining product purity and morphology, with ammonium phosphate producing Ag_3_PO_4_ of the highest phase purity, while the introduction of alcohol into the reaction medium significantly reduced the particle size by suppressing crystal growth and agglomeration [11]. However, despite the promising phytochemical accumulation and antioxidant activity of Ag_3_PO_4_ in hydroponic systems, it remains largely unexplored.

Addressing this critical knowledge gap, the present study extends the application of Ag_3_PO_4_ to a practical hydroponic cultivation system to evaluate plant growth, chlorophyll content, phytochemical accumulation and antioxidant activity under conditions that closely resemble commercial production. Therefore, this work aims to synthesize Ag_3_PO_4_ and evaluate the effects of Ag_3_PO_4_-derived supplementation on plant growth, chlorophyll content, phytochemical accumulation and antioxidant activity in *C. albida*. This study provides fundamental information on the effects of Ag_3_PO_4_-derived supplementation on the physiological and biochemical responses of *C. albida* under hydroponic cultivation. The findings may contribute to the development of cultivation strategies for improving phytochemical accumulation and antioxidant activity in high-value aquatic plants, thereby supporting the production and export of ornamental aquatic species.

## 2. Results and Discussion

### 2.1. Synthesis and Characterization of Ag_3_PO_4_

#### 2.1.1. Fourier Transform Infrared Spectroscopy (FTIR)

**Figure 1:**
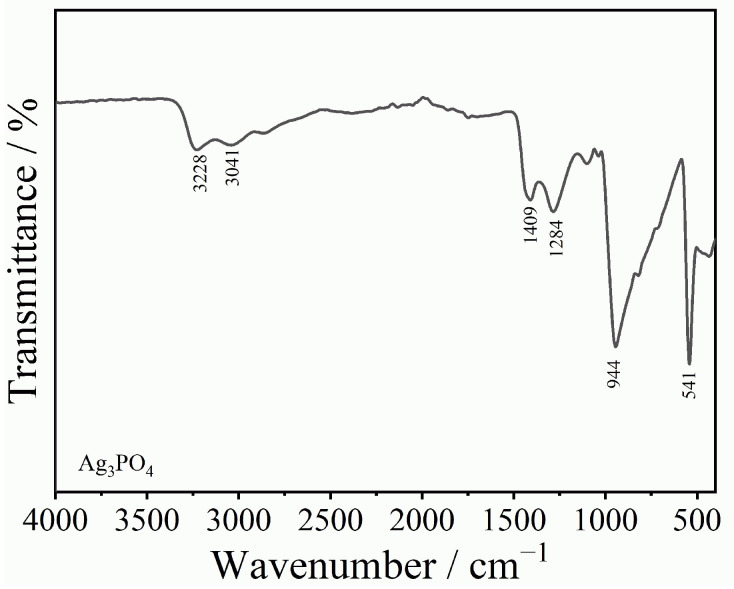
FTIR spectrum of silver phosphate (Ag_3_PO_4_).

The FTIR spectrum of silver phosphate (Ag_3_PO_4_) identified by Fourier Transform Infrared Spectroscopy (FTIR) is shown in Figure 1. The FTIR spectrum exhibits characteristic peaks associated with the phosphate (PO_4_^3−^) group, confirming the successful formation of silver phosphate (Ag_3_PO_4_) in the sample. The strong peaks at 944 and 541 cm^−1^ are assigned as P-O stretching and O-P-O bending of the PO_4_^3−^ tetrahedral unit, respectively [16]. The bands observed at 1409 and 1284 cm^−1^ are attributed to the P=O stretching vibrations within the PO_4_^3−^ group [17]. In addition, the broad absorption band between around 3400 and 2900 cm^−1^ corresponds to O-H stretching of surface water molecules. These characteristic peaks are similar to the FTIR spectrum of silver phosphate synthesized by Masaoudi et al. [18]. A distinct absorption band at 1284 cm^−1^ is also related to the bending vibration of H–O–H in adsorbed water. However, it does not correspond to the conventional bending mode but could be assigned to a low-frequency H–O–H bending vibration associated with strongly hydrogen-bonded water species on the particle surface [19,20].

#### 2.1.2. X-Ray Diffraction (XRD)

**Figure 2:**
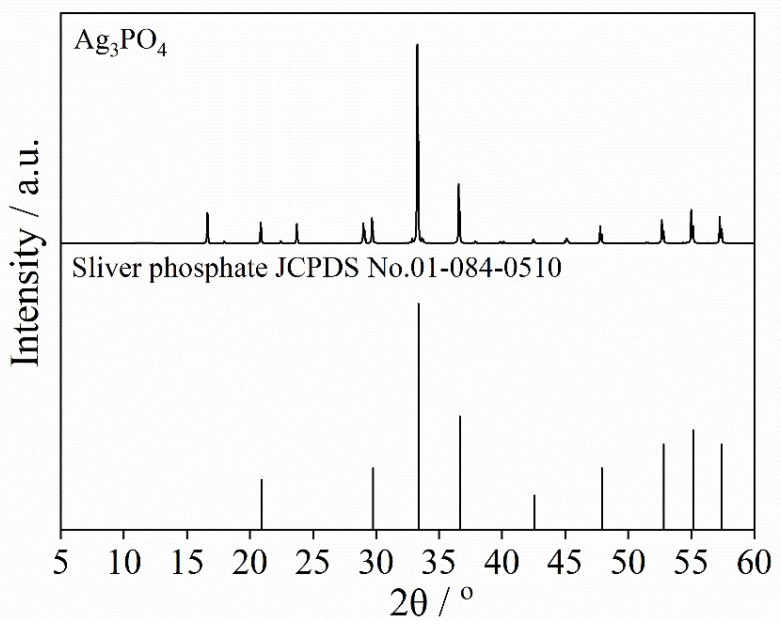
XRD pattern of silver phosphate (Ag_3_PO_4_) compared to standard JCPDS no. 01-084-0510.

The crystallinity and phase structure of the synthesized silver phosphate (Ag_3_PO_4_) analyzed by the X-ray diffraction (XRD) technique was shown in Figure 2. As shown in Figure 2 the major diffraction peaks of the sample were consistent with the standard diffraction data of cubic Ag_3_PO_4_, corresponding to the JCPDS database (No. 01-084-0510), which exhibits relatively intense peaks at 20.88°, 29.69°, 33.30°, 36.58°, 42.49°, 47.80°, 52.69°, 55.02° and 57.28°, corresponding to the (110), (200), (210), (211), (220), (310), (222), (320) and (321) crystal planes of cubic Ag_3_PO_4_, respectively. The average crystallite sizes (S_(c)_ of the synthesized silver phosphate (Ag_3_PO_4_) were estimated using the Scherrer equation, as expressed in Equation (1). Based on this analysis, the crystallite sizes were determined to be 123.68 nm.

(1) $$Sc=\frac{0.94\lambda }{\beta \;cos\theta }$$

where λ is the employed X-ray wavelength (0.154059 nm), β is the full width at the half maximum (FWHM in radians) of each investigated diffraction peak, and θ is the diffraction peak angle.

Additionally, other intense peaks are observed at 16.5°, 23.6°, 28.8°, and 45.1°. Previous studies have demonstrated that the pH of the reaction medium and the choice of phosphate precursor can significantly influence the crystallographic outcome, leading not only to the formation of Ag_3_PO_4_ but also to other silver-phosphate-related phases under certain conditions, as reported by Tóth et al. [21]. It was observed that when NaH_2_PO_4_ was used as the phosphate source, the diffraction patterns exhibited additional reflections at approximately 2θ ≈ 27.0°, 30.6°, and 32.1°, which were identified as characteristic peaks of silver pyrophosphate (Ag_4_P_2_O_7_). The authors attributed this to the relatively higher proton concentration and subsequent phosphate condensation in less basic environments, which favor pyrophosphate formation alongside Ag_3_PO_4_.

#### 2.1.3. X-Ray Fluorescence (XRF)

X-ray fluorescence (XRF) spectroscopy was employed as a reliable analytical method for both qualitative and quantitative determination of the elemental composition of the Ag_3_PO_4_ sample. The XRF results, expressed as oxide-equivalent compositions, showed 86.80% of Ag_2_O and 12.60% of P_2_O_5_ as the major components, with an Ag:PO_4_ mole ratio of 3.21:1.00. About 0.58% of minor components are also detected, with less than 0.6 wt%, including 0.075% of SiO_2_, 0.415% of SO_3_, 0.033% of Cl, and 0.056% of PdO.

#### 2.1.4. Field Emission–Scanning Electron Microscope (FE-SEM)

**Figure 3:**
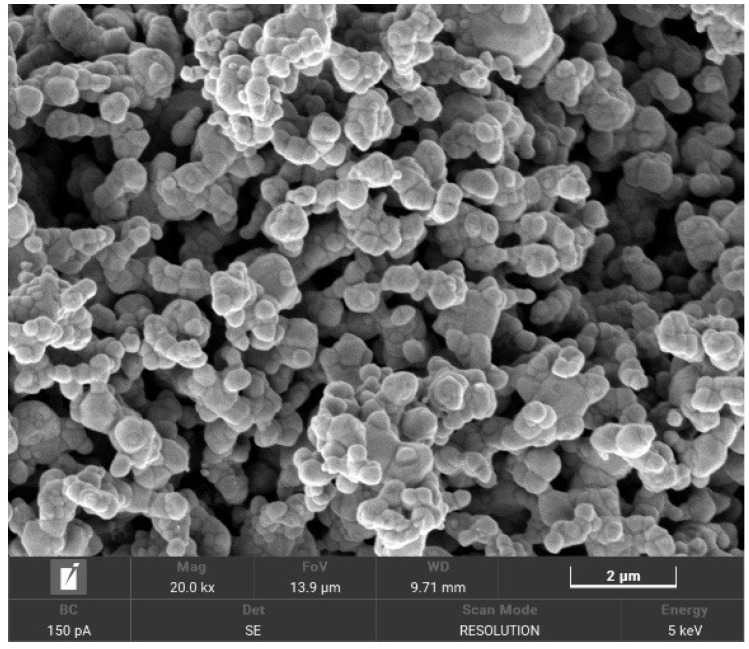
Morphology of silver phosphate (Ag_3_PO_4_).

The morphology of synthesized silver phosphate (Ag_3_PO_4_) analyzed by a field emission–scanning electron microscope (FE-SEM) is shown in Figure 3. The FE-SEM image of Ag_3_PO_4_ exhibits spherical-shaped particles with different sizes between 300 and 500 nm. In previous research, Tóth et al. [21] precipitated silver phosphate microparticles (Ag_3_PO_4_) using only water as a medium, resulting in spherical particles of silver phosphate with average particle sizes of around 1.5 µm. Febiyanto et al. [22] precipitated silver phosphate (Ag_3_PO_4_) using 25% ammonia solution as a medium, and the SEM images exhibit the sphere-like particles with sizes around 0.5–2 µm. The particle size of materials is a critical parameter that directly influences their physicochemical properties and, consequently, their practical applications. Precise control over particle size is therefore of considerable importance, as it significantly affects surface area, reactivity, and overall material performance. In this context, the use of a water–ethanol mixture at a 50:50 volume ratio for the precipitation of silver phosphate in the present study demonstrates considerable potential for effectively controlling crystal growth and reducing the particle size of the resulting compound.

### 2.2. Effects of Silver Phosphate Supplementation on the Growth and Antioxidant Activity of Cryptocoryne albida Parker in Hydroponics

#### 2.2.1. Plant Growth

**Figure 4:**
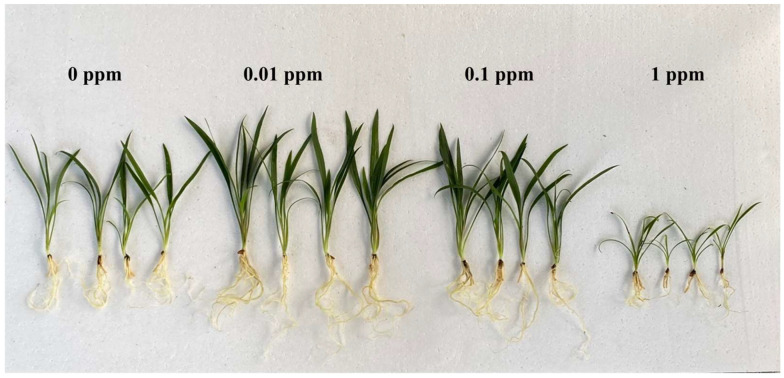
*C. albida* cultured in KMITL2 nutrient solution, adding Ag_3_PO_4_-derived supplementation at 0, 0.01, 0.10, and 1.0 ppm under DFT hydroponics.

The results of the growth study of *C. albida* supplemented with Ag_3_PO_4_-derived preparation at different concentrations of 0.00, 0.01, 0.10, and 1.00 ppm in DFT hydroponics showed that the growth of the plant was significantly different (*p* < 0.05) after 8 weeks of cultivation. The growth performance, including plant height, leaf length, leaf number, leaf width, root number, and root length, was significantly lower in plants grown in a nutrient solution with 1.00 ppm added than in the other treatments (Figure 4 and Table 1). Plant growth performance did not show significant differences among plants grown in the KMITL 2 nutrient solution with 0.01 and 0.10 ppm Ag_3_PO_4_ and the control. Plant growth performance significantly decreased with increasing concentration of Ag_3_PO_4_-derived supplementation (*p* < 0.05). A similar experiment by Al-Huqail et al. [23] conducted Ag supplementation at 0, 0.1, 0.3, and 0.5 mg/L to *Lupinus termis* L. seedlings for ten days. It was found that the Ag concentration of 0.1 mg/L led to a significant increase in seedling growth (root and shoot lengths, fresh and dry weights). Additionally, *C. albida* treated with 1 ppm Ag_3_PO_4_-derived supplementation exhibited a shorter root length than other treatments (*p* < 0.05). This agrees with Al-Huqail et al. [23] who reported that adding 0.3 and 0.5 mg/L of Ag in germination of *L. termis* L. led to a reduction in root and shoot length and biomass. Similarly, this was observed when testing three Ag sizes (20, 40, and 80 nm) and four dosage concentrations (66.84, 133.68, 267.36, and 534.72 mg/L) for *Arabidopsis thaliana* seeding and germination. It was found that exposure of plant roots to Ag resulted in the root tips turning brown [24]. This is consistent with the research of Vinkovic et al. [25], who experimented with Ag on sweet pepper plants (*Capsicum annuum* L.) at different concentrations of 0.01, 0.05, 0.1, and 1 mg/L. Ag was found to have a concentration of 0.01 mg/L. It also has the highest plant height. Plant growth inhibition varies significantly with nanoparticle size and plant variety. Ag with higher concentration levels results in extreme toxicity, and Ag in which the particles are very small exceeds the toxicity of plants [24,26,27].

#### 2.2.2. Chlorophyll Contents in *C. albida*

Table 2 shows that chlorophyll a, chlorophyll b, and total chlorophyll contents of *C. albida* differed significantly among treatments (*p* < 0.05). The lowest values of all chlorophyll parameters were observed in plants treated with 1.00 ppm Ag_3_PO_4_-derived supplementation, whereas no significant differences were found among the control, 0.01 ppm, and 0.10 ppm treatments. These results indicate that low concentrations of Ag_3_PO_4_-derived supplementation did not significantly affect chlorophyll accumulation, while the highest concentration tested exerted an inhibitory effect.

Similar reductions in chlorophyll content at elevated silver concentrations have been reported in Arabidopsis thaliana exposed to Ag under hydroponic conditions [28]. Likewise, Li et al. [29] demonstrated that exposure to Ag could influence chlorophyll metabolism in A. thaliana, indicating that silver-containing materials may affect photosynthetic pigments depending on the exposure conditions. However, plant responses to silver-based materials vary considerably among species and concentrations. For example, increased chlorophyll content has been observed in *Hordeum vulgare* L. and *Silybum marianum* under certain Ag concentrations, whereas chlorophyll reduction has been reported in *Dracocephalum moldavica* at higher exposure levels [30,31]. These findings suggest that the effects of silver-containing materials on chlorophyll biosynthesis are species-dependent and influenced by both the concentration and physicochemical properties of the silver source.

#### 2.2.3. Antioxidant Properties in *C. albida*

Table 3 shows that supplementation with 0.01 ppm Ag_3_PO_4_ resulted in the highest TPC and TFC values in *C. albida*, while DPPH and ABTS activities were significantly enhanced at both 0.01 and 0.10 ppm compared with the control and 1.00 ppm treatments. These findings indicate that the physiological response of *C. albida* to Ag_3_PO_4_-derived supplementation was strongly concentration-dependent, with low concentrations promoting phytochemical accumulation and antioxidant activity, whereas higher concentrations reduced these beneficial effects. The observed response may reflect a mild stress-induced stimulation of secondary metabolite production associated with low concentrations of the Ag_3_PO_4_-derived preparation, while excessive exposure may negatively affect plant metabolism. Similar concentration-dependent responses have been reported for other silver-containing materials, including AgNPs, where low concentrations enhanced phenolic compounds, flavonoids, and antioxidant activities in plants [32,33,34]. However, because the dissolution behavior and silver speciation of Ag_3_PO_4_ were not investigated in the present study, direct comparisons with AgNPs should be interpreted cautiously. Therefore, the results primarily demonstrate the physiological and biochemical responses of *C. albida* to Ag_3_PO_4_-derived supplementation under the hydroponic conditions evaluated in this work.

## 3. Materials and Methods

### 3.1. Synthesis and Characterization of Ag_3_PO_4_

#### 3.1.1. Starting Materials Preparation

All chemicals used in this study, including silver nitrate (AgNO_3_, 99%, Merck, Darmstadt, Germany), diammonium hydrogen phosphate ((NH_4_)_2_HPO_4_, 99%, KemAus, Adelaide, Australia), and ethyl alcohol (C_2_H_5_OH, 99%, Q RëC^TM^, Auckland, New Zealand), were analytical reagent (AR) grade and used without further purification. In this study, a mixed solvent was employed to dissolve all precursor chemicals. The solvent was prepared by combining ethyl alcohol with deionized water at an equal volume ratio of 1:1. Silver nitrate (AgNO_3_) and diammonium hydrogen phosphate ((NH_4_)_2_HPO_4_) were individually dissolved in the prepared solvent to produce precursor solutions with a concentration of 0.20 mol·L^−1^, which were subsequently used for the synthesis of Ag_3_PO_4_.

#### 3.1.2. Synthesis of Silver Phosphate (Ag_3_PO_4_)

A volume of 300 mL of the 0.2 mol·L^−1^ silver nitrate solution was transferred into a beaker as a silver cations (Ag^+^) source, followed by the slow addition of 100 mL of the 0.2 mol·L^−1^ diammonium hydrogen phosphate solution as a phosphate anions source (PO_4_^3−^), corresponding to the stoichiometric mole ratio of Ag^+^ cations per PO_4_^3−^ anions of 3:1 formed as silver phosphate (Ag_3_PO_4_) according to Equation (1), while continuously stirring at 400 rpm using a magnetic stirrer for 30 min. After that, the yellow suspension was precipitated. The obtained suspension was filtered through filter paper (Whatman, No. 42, Cytiva, Marlborough, MA, USA) and washed with ethyl alcohol three times. The obtained solid was dried in an oven at 60 °C for 2h.

3 Ag^+^ (aq) + PO_4_^3−^ (aq) → Ag_3_PO_4_ (s) (2)

For further supplementation of the growth and antioxidant activity of *Cryptocoryne albida* in hydroponic applications, silver phosphate (Ag_3_PO_4_) was prepared as a stock solution. Due to the limited solubility of Ag_3_PO_4_ in water, acetic acid was used during stock solution preparation. The resulting solution was subsequently diluted with deionized water to obtain a stock solution with a Ag_3_PO_4_ concentration of 100 ppm. The chemical speciation of silver in the final nutrient solution was not determined in the present study. Therefore, the supplemented preparation may contain particulate and/or dissolved silver species.

#### 3.1.3. Characterization of Silver Phosphate (Ag_3_PO_4_)

The functional groups present in the synthesized silver phosphate sample were investigated using Fourier transform infrared (FTIR) spectroscopy (Spectrum GX, PerkinElmer, Waltham, MA, USA). The FTIR spectra were obtained using the KBr pellet method under a wavenumber range of 4000–400 cm^−1^, with 8 scans and a spectral resolution of 4 cm^−1^. The crystal structure and phase purity of the samples were examined by X-ray diffraction (XRD) using a Rigaku MiniFlex diffractometer (Rigaku, The Woodlands, TX, USA) equipped with Cu–Kα radiation (λ = 0.15406 nm). Diffraction patterns were recorded over the 2θ range of 5–60° with a step size of 0.01° and a scan rate of 1 s per step, using an accelerating voltage of 30 kV and a current of 40 mA. The obtained diffraction data were compared with standard reference patterns from the Joint Committee on Powder Diffraction Standards (JCPDS) database to identify the crystalline phases. The surface morphology of the samples was analyzed using a field emission scanning electron microscope (FESEM, LEO VP1450, Zeiss, Jena, Germany) operated at an accelerating voltage of 15 kV. Prior to FESEM observation, the samples were sputter-coated with a thin layer of gold to enhance electrical conductivity and minimize charging effects. The elemental composition of the samples was determined by X-ray fluorescence spectroscopy (XRF), using a Supermini200 spectrometer (Rigaku, The Woodlands, TX, USA).

### 3.2. Effects of Silver Phosphate Supplementation on the Growth and Antioxidant Activity of Cryptocoryne albida Parker in Hydroponics

#### 3.2.1. Experimental Design

The experimental design was completely randomized, with Ag_3_PO_4_ added at 4 concentration levels (0 ppm [control group], 0.01 ppm, 0.10 ppm, and 1.0 ppm) into the KMITL 2 nutrient solution. Each treatment has 4 replications, with 10 plants per replicate.

#### 3.2.2. Hydroponics Culture System Preparation

Prepare a DFT planting system using white PVC pipes, 1 m long and 55 mm in diameter, with 38 mm holes drilled, including 10 holes for recirculating the nutrient solution. The plant was grown under greenhouse conditions with natural light, temperature (30–32 °C), and humidity (70–80%), using an automatic water-spray system that sprayed water every 10 min for 20 s.

#### 3.2.3. Aquatic Plant Material and Cultivation

Selected *C. albida* from the aquatic plant greenhouse of the faculty of agricultural technology, King Mongkut’s Institute of Technology Ladkrabang (KMITL), Bangkok, Thailand. The *C. albida* were separated into single plants, and the roots and leaves were cut to the same size on all plants. Selected *C. albida* of similar size were planted in the prepared DFT system. Using a nutrient solution formula, KMITL 2 was prepared according to Nantagit methods [35], containing 12.20 mM NO_3_^−^, 1.20 mM H_2_PO_4_^−^, 1.00 mM SO_4_^2−^, 5.36 mM K^+^, 3.72 mM Ca^2+^ and 1.00 mM Mg^2+^, while the micronutrient concentration was: 55.00 µM Fe, 35.00 µM B_2_O_3_, 20.00 µM Mn, 4.00 µM Zn, 1.00 µM Cu, and 0.50 µM Mo. The nutrient solution concentration was 0.5 mS/cm, and then four levels of Ag_3_PO_4_-derived preparation (0, 0.01, 0.10, and 1.00 ppm, based on the prepared stock solution) were added. The pH of the nutrient solution was adjusted to between 6.5 and 7.5 using KOH and HNO_3_ at 10%. The EC and pH values are monitored weekly, and the nutrient solution is replaced in the 4th week.

#### 3.2.4. Measurements and Data Collections

Growth performance was recorded every 2 weeks for 8 weeks. Five plants were randomly selected from each replicate for measurement. The mean value obtained from the five plants was used to represent each replicate for subsequent statistical analysis. The growth and changes in the *C. albida* were measured including the number of leaves, plant height, leaf length (from the tip of the leaf to the base of the leaf), leaf width (in the middle of the leaf, the widest part) by length and width of the leaf are measured at the 3rd leaf. At the end of the experiment, the number and length of the roots are measured. The amount of chlorophyll content in the leaves of the *C. albida* according to the method of EPA [36], and the analysis of its antioxidant potential. For phytochemical and antioxidant analyses, oven-dried plant samples collected at harvest were ground into powder. A sample (0.20 g dry weight) was extracted with 20 mL of 95% ethanol at room temperature for 24 h. The extracts were filtered through Whatman No. 1 filter paper, adjusted to the original volume, and stored in amber bottles at 4 °C until analysis.

The total phenolic content (TPC) was determined using the Folin–Ciocalteu method [37]. Briefly, 100 μL of extract was mixed with 500 μL distilled water and 100 μL Folin–Ciocalteu reagent. After 6 min, 1 mL of 7% Na_2_CO_3_ and 500 μL distilled water were added. The mixture was incubated for 90 min at room temperature, and absorbance was measured at 760 nm using a UV–Vis spectrophotometer (UV-1800, Shimadzu, Kyoto, Japan). Gallic acid was used as the calibration standard.

The total flavonoid content (TFC) was determined using the aluminum chloride colorimetric method. Briefly, 50 μL of extract was mixed with 100 μL of 10% AlCl_3_, followed by 1 mL of 1 M potassium acetate and 2.8 mL distilled water. The mixture was incubated for 30 min at room temperature and absorbance was measured at 415 nm. Quercetin was used as the calibration standard.

The DPPH radical scavenging activity was determined according to Shirazi et al. [37]. Briefly, 50 μL of extract was mixed with 3 mL of 0.002% DPPH solution and incubated in the dark for 15 min. Absorbance was measured at 515 nm, and scavenging activity was calculated as the percentage of inhibition relative to the control.

ABTS radical solution was prepared by reacting 7 mM ABTS with 140 mM potassium persulfate and incubating the mixture in the dark for 16 h. The resulting solution was diluted with ethanol before use. Extract (300 μL) was mixed with 2.7 mL of diluted ABTS solution and incubated in the dark for 20 min. Absorbance was measured at 734 nm, and scavenging activity was expressed as the percentage of inhibition relative to the control.

#### 3.2.5. Statistical Data Analysis

Each hydroponic cultivation unit (replicate) was considered an experimental unit. For each replicate, five plants were randomly selected for measurement, and the mean value of these plants was used for subsequent statistical analysis. All data were statistically analyzed using analysis of variance (ANOVA) and Duncan’s New Multiple Range Test (DMRT) to determine differences among treatments at the 0.05 alpha level of confidence. Statistical analyses were performed using IBM SPSS Statistics V.29 with a license from King Mongkut’s Institute of Technology Ladkrabang (Server IP: 10.252.92.33). All results are presented as mean ± standard error (SE).

## 4. Conclusions

Silver phosphate (Ag_3_PO_4_) was successfully synthesized via a simple precipitation route using AgNO_3_ and (NH_4_)_2_HPO_4_, with an alcohol as the reaction medium to control particle growth and agglomeration. FTIR analysis confirmed the presence of characteristic phosphate functional groups, while XRD patterns indicated Ag_3_PO_4_ as the dominant crystalline phase in the synthesized material. XRF results exhibit 86.80% of Ag_2_O and 12.60% of P_2_O_5_ as the major components, and SEM observations revealed relatively uniform spherical particles with sizes ranging from approximately 300 to 500 nm. The use of ammonium phosphate as the phosphate source and alcohol-assisted synthesis contributed to the formation of Ag_3_PO_4_ particles with a uniform morphology compared with previous reports. When applied in a DFT hydroponic system, supplementation with an Ag_3_PO_4_-derived preparation at a low concentration (0.01 ppm) was associated with significantly higher phytochemical accumulation, including TPC, TFC, and antioxidant activities (DPPH and ABTS), in *C. albida*. In contrast, low supplementation concentrations (0.01 and 0.10 ppm) did not significantly affect plant growth or chlorophyll content compared with the control, whereas 1.00 ppm significantly reduced these parameters. Overall, the findings provide preliminary information on the physiological and biochemical responses of *C. albida* to Ag_3_PO_4_-derived supplementation under hydroponic cultivation conditions. The present study has several limitations. The chemical speciation of silver, dissolved Ag concentration, particle stability in the nutrient solution, residual Ag and P accumulation in plant tissues, and possible analytical interference in the antioxidant assays were not determined. Therefore, the observed responses should be interpreted as physiological and biochemical effects associated with Ag_3_PO_4_-derived supplementation under the tested hydroponic conditions, rather than evidence of a defined particle-specific mechanism.

## Figures and Tables

**Table 1:** Growth performance of *C. albida* cultured in experimental nutrient solutions containing different concentrations of Ag_3_PO_4_-derived supplementation.

Parameters	Ag_3_PO_4_-Derived Supplementation (ppm)				F-Test
	0.00	0.01	0.10	1.00	
Plant height (cm.)	13.72 ± 2.56 ^a^	15.39 ± 0.46 ^a^	14.43 ± 1.51 ^a^	8.30 ± 1.16 ^b^	*
Leaves length (cm.)	8.29 ± 1.32 ^a^	9.26 ± 0.37 ^a^	8.42 ± 1.07 ^a^	4.17 ± 1.02 ^b^	*
Leaves width (cm.)	0.74 ± 0.11 ^a^	0.85 ± 0.02 ^a^	0.82 ± 0.10 ^a^	0.43 ± 0.06 ^b^	*
Leaves number (no./plant)	6.40 ± 1.24 ^a^	7.21 ± 0.60 ^a^	6.88 ± 1.07 ^a^	4.30 ± 1.24 ^b^	*
Root length (cm.)	19.72 ± 2.03 ^a^	21.22 ± 1.97 ^a^	19.30 ± 2.07 ^a^	12.04 ± 1.99 ^b^	*
Roots number (no./plant)	5.55 ± 0.42 ^a^	6.06 ± 0.23 ^a^	5.79 ± 0.46 ^a^	4.04 ± 0.25 ^b^	*

* = significant at *p* ≤ 0.05. Mean ± SE with different letters in the same row represent statistically significant differences according to DMRT (*p* ≤ 0.05).

**Table 2:** Chlorophyll content of *C. albida* cultured in experimental nutrient solutions containing different concentrations of Ag_3_PO_4_-derived supplementation.

Ag_3_PO_4_-Derived Supplementation (ppm)	Chlorophyll a (mg/g)	Chlorophyll b (mg/g)	Total Chlorophyll Content (mg/g)
0.00	0.82 ± 0.27 ^a^	0.27 ± 0.06 ^a^	1.09 ± 0.40 ^a^
0.01	0.60 ± 0.26 ^a^	0.27 ± 0.10 ^a^	0.87 ± 0.37 ^a^
0.10	0.59 ± 0.17 ^a^	0.21 ± 0.16 ^ab^	0.80 ± 0.27 ^a^
1.00	0.25 ± 0.09 ^b^	0.12 ± 0.13 ^b^	0.36 ± 0.15 ^b^
F-test	*	*	*

* = significant at *p* ≤ 0.05. Mean ± SE with different letters in the same column represent statistically significant differences according to DMRT (*p* ≤ 0.05).

**Table 3:** Antioxidant properties of *C. albida-*cultured experimental nutrient solutions contained different concentrations of Ag_3_PO_4_-derived supplementation.

Ag_3_PO_4_-Derived Supplementation (ppm)		Phytochemicals		
	TPC (mgGAE/g)	TFC (mgQE/g)	DPPH (% Inhibition)	ABTS (% Inhibition)
0.00	0.94 ± 0.09 ^b^	0.94 ± 0.11 ^b^	4.59 ± 0.69 ^b^	25.28 ± 2.05 ^b^
0.01	1.81 ± 0.08 ^a^	3.83 ± 0.11 ^a^	7.58 ± 0.97 ^a^	55.45 ± 7.84 ^a^
0.10	0.94 ± 0.11 ^b^	0.94 ± 0.39 ^b^	7.53 ± 0.78 ^a^	47.76 ± 7.57 ^a^
1.00	0.90 ± 0.11 ^b^	0.88 ± 0.18 ^b^	2.70 ± 0.77 ^b^	10.74 ± 2.34 ^b^
F-test	*	*	*	*

* = significant at *p* ≤ 0.05. Mean ± SE with different letters in the same column represent statistically significant differences according to DMRT (*p* ≤ 0.05).

## Data Availability

The original contributions presented in this study are included in the article. Further inquiries can be directed to the corresponding authors.
